# Functional Hierarchy of Uterotonics Required for Successful Parturition in Mice

**DOI:** 10.1210/en.2019-00499

**Published:** 2019-09-13

**Authors:** Masahide Yoshida, Yuki Takayanagi, Azusa Ichino-Yamashita, Kei Sato, Yukihiko Sugimoto, Tadashi Kimura, Katsuhiko Nishimori

**Affiliations:** 1 Laboratory of Molecular Biology, Department of Molecular and Cell Biology, Graduate School of Agricultural Science, Tohoku University, Sendai-shi, Miyagi-ken, Japan; 2 Division of Brain and Neurophysiology, Department of Physiology, Jichi Medical University, Shimotsuke-shi, Tochigi-ken, Japan; 3 Division of Systems Virology, Institute of Medical Science, The University of Tokyo, Minato-ku, Tokyo-to, Japan; 4 CREST, Japan Science and Technology Agency, Kawaguchi-shi, Saitama-ken, Japan; 5 Department of Pharmaceutical Biochemistry, Graduate School of Pharmaceutical Sciences, Kumamoto University, Chuo-Ku, Kumamoto-ken, Japan; 6 Department of Obstetrics and Gynecology, Osaka University Graduate School of Medicine, Suita-shi, Osaka-hu, Japan; 7 Department of Obesity and Inflammation Research, Fukushima Medical University, Fukushima-shi, Fukushima-ken, Japan; 8 Department of Bioregulation and Pharmacological Medicine, Fukushima Medical University, Fukushima-shi, Fukushima-ken, Japan

## Abstract

Parturition is an essential process in placental mammals for giving birth to offspring. However, the molecular machineries of parturition are not fully understood. We investigated whether oxytocin plays a crucial role in the progress of parturition in cooperation with the prostaglandin F_2*α*_ (PGF_2*α*_) receptor. We first examined alterations in the expression of uterine contraction-associated genes in uteri of oxytocin receptor–deficient mice (*Oxtr*^−/−^) during parturition. We found that induction of cyclooxygenase (COX)-2 and connexin 43 expression was impaired in *Oxtr*^*−/−*^, whereas that of PGF_2*α*_ receptor expression was not. We next generated mice with double knockout of genes for the oxytocin receptor/oxytocin and PGF_2*α*_ receptor (*Oxtr*^*−/−*^;*Ptgfr*^*−/−*^ and *Oxt*^*−/−*^;*Ptgfr*^*−/−*^) and evaluated their parturition with *Oxtr*^*−/−*^, *Oxt*^*−/−*^, *Ptgfr*^*−/−*^, and wild-type mice. In *Oxtr*^*−/−*^;*Ptgfr*^*−/−*^ and *Oxt*^*−/−*^;*Ptgfr*^*−/−*^, pregnancy rates were similar to those of other genotypes. However, normal parturition was not observed in *Oxtr*^*−/−*^;*Ptgfr*^*−/−*^ or *Oxt*^*−/−*^;*Ptgfr*^*−/−*^ because of persistent progesterone from the corpus luteum, as observed in *Ptgfr*^*−/−*^. We administered RU486, a progesterone antagonist, to *Ptgfr*^*−/−*^, *Oxtr*^*−/−*^;*Ptgfr*^*−/−*^, and *Oxt*^*−/−*^;*Ptgfr*^*−/−*^ on gestation day 19. These mice were able to deliver a living first pup and the parturition onset was similar to that in *Ptgfr*^*−/−*^. Meanwhile, unlike *Ptgfr*^*−/−*^, ∼75% of *Oxtr*^*−/−*^;*Ptgfr*^*−/−*^ and *Oxt*^*−/−*^;*Ptgfr*^*−/−*^ administered RU486 remained in labor at 24 hours after the onset of parturition. All of the pups that experienced prolonged labor died. We thus revealed that the oxytocin receptor is an upstream regulator of COX-2 and connexin 43 in the uterus during parturition and that both oxytocin/oxytocin receptor and PGF_2*α*_ receptor are major components for successful parturition.

The neurohypophysial hormone oxytocin, which is named after the Greek words meaning “quick birth,” is primarily synthesized in the hypothalamus and secreted mainly from the posterior pituitary into systemic circulation. Oxytocin has been long known as one of the most potent uterotonic factors. Both the secretion of oxytocin from the pituitary into blood and the expression of the oxytocin receptor in the uterus are coordinately upregulated around the time of parturition, and oxytocin has thus been used to induce or augment labor in clinical practice (1). Although it was thought that oxytocin/oxytocin receptor signaling was essential for parturition in mammals, oxytocin-deficient mice (*Oxt*^*−/−*^) and oxytocin receptor–deficient mice (*Oxtr*^*−/−*^) showed normal parturition, and roles of the oxytocin system in parturition remain unclear (2, 3).

Vasopressin, the other nonapeptide hormone synthesized in the hypothalamus, is also secreted from the posterior pituitary. It was shown that vasopressin neurons in the supraoptic nucleus of the hypothalamus are activated during parturition (4) and that vasopressin release from the posterior pituitary is enhanced (5). It was also shown that vasopressin stimulates uterine contraction not via the vasopressin receptor but via the oxytocin receptor in late pregnancy in mice (3, 6) and that sensitivity to vasopressin increased at late pregnancy (7). Thus, vasopressin has also been expected to act as a uterotonic factor during parturition.

Prostaglandins have luteolytic and uterotonic actions in parturition (8). Prostaglandin F_2*α*_ (PGF_2*α*_) and prostaglandin E_2_ (PGE_2_) are known as uterotonic factors and have been clinically used to augment labor (8). Parturition in rodents is preceded by a decline of maternal blood progesterone concentration via luteolysis, which leads to induction of myometrial contractility. In PGF_2*α*_ receptor–deficient mice (*Ptgfr*^*−/−*^), progesterone withdrawal in blood during late pregnancy and the subsequent onset of parturition did not occur. A reduction of blood progesterone level with ovariectomy at day 19 of pregnancy induced expression of the oxytocin receptor in the uteri and enabled successful parturition in *Ptgfr*^*−/−*^, although uterine contractile activity via the PGF_2*α*_ receptor was lost (9). PGE_2_ also induces contraction in the pregnant myometrium (10). Cyclooxygenase (COX)-1 and COX-2, which convert arachidonic acid into prostaglandin H_2_, are expressed in the uterus during pregnancy. Expression of COX-2 is upregulated and COX-2 provides PGE_2_ and PGF_2*α*_ to the uterine myometrium during parturition (8, 11). Inhibition of the enzymatic activity of COX-2 prevented inflammation-mediated preterm birth in mice (12). Uterotonic actions of PGE_2_ were mediated via the EP3 receptor (13, 14). However, EP3 receptor–deficient mice showed normal parturition (15).

Gap junctions are plasma membrane domains containing intercellular channels that can exchange ions, second messengers, and small metabolites between neighboring cells. An intercellular gap junction is composed of connexin proteins (16). The expression of connexin 43, a major myometrial gap junction protein, significantly increases just before the onset of parturition in both rats and humans (17). It has been suggested that enhancing connectivity among myometrial cells via connexin 43 is essential for synchronized contractions required to expel the fetus. A loss-of-function connexin 43 mutant reduced uterine contraction in response to oxytocin, indicating that connexin 43 sensitizes myometrial cells to oxytocin (18). Although conditional knockout mice with smooth muscle–specific ablation of connexin 43 showed prolonged gestation, the mice were able to deliver their pups (19).

To clarify the hierarchy of these molecules for preparation, onset, and progress of parturition, we first examined the expression of mRNA for uterine contraction-associated genes in the uterus of *Oxtr*^*−/−*^ during parturition. We also generated mice with double knockout of the genes for the oxytocin receptor and PGF_2*α*_ receptor and mice with double knockout of the genes for oxytocin and the PGF_2*α*_ receptor (*Oxtr*^*−/−*^;*Ptgfr*^*−/−*^ and *Oxt*^*−/−*^;*Ptgfr*^*−/−*^) and evaluated their parturition.

## Materials and Methods

### Maintenance of mice

Animal experiments were carried out after receiving approval from the Animal Experiment Committee of Tohoku University and were conducted in accordance with the Institutional Regulations for Animal Experiments and Fundamental Guidelines for Proper Conduct of Animal Experiments and Related Activities in Academic Research Institutions under the jurisdiction of the Ministry of Education, Culture, Sports, Science, and Technology of Japan.

We used *Oxtr*^*−/−*^, *Oxt*^*−/−*^, and *Ptgfr*^*−/−*^ with a chimeric background (129 × C57BL/6J), generated previously (2, 3, 9). *Oxtr*^*−/−*^;*Ptgfr*^*−/−*^ and *Oxt*^*−/−*^;*Ptgfr*^*−/−*^ were produced by intercrosses. A 1:2:1:2:4:2:1:2:1 Mendelian distribution of the progeny from *Oxtr*^*+/−*^;*Ptgfr*^*+/−*^ or *Oxt*^*+/−*^;*Ptgfr*^*+/−*^ intercrosses was observed [(*Oxtr*^*−/−*^;*Ptgfr*^*−/−*^, *Oxtr*^*+/−*^;*Ptgfr*^*−/−*^, *Oxtr*^*+/+*^;*Ptgfr*^*−/−*^, *Oxtr*^*−/−*^;*Ptgfr*^*+/−*^, *Oxtr*^*+/−*^;*Ptgfr*^*+/−*^, *Oxtr*^*+/+*^;*Ptgfr*^*+/−*^, *Oxtr*^*−/−*^;*Ptgfr*^*+/+*^, *Oxtr*^*+/−*^;*Ptgfr*^*+/+*^, *Oxtr*^*+/+*^;*Ptgfr*^*+/+*^), 5:26:15:19:39:28:10:26:5; (*Oxt*^*−/−*^;*Ptgfr*^*−/−*^, *Oxt*^*+/−*^;*Ptgfr*^*−/−*^, *Oxt*^*+/+*^;*Ptgfr*^*−/−*^, *Oxt*^*−/−*^;*Ptgfr*^*+/−*^, *Oxt*^*+/−*^;*Ptgfr*^*+/−*^, *Oxt*^*+/+*^;*Ptgfr*^*+/−*^, *Oxt*^*−/−*^;*Ptgfr*^*+/+*^, *Oxt*^*+/−*^;*Ptgfr*^*+/+*^, *Oxt*^*+/+*^;*Ptgfr*^*+/+*^), 16:48:21:41:62:51:17:42:20]. This result indicated that double deletion of oxytocin or the oxytocin receptor and the PGF_2*α*_ receptor did not affect embryonic lethality. Body weights of wild-type (WT), *Oxtr*^*−/−*^;*Ptgfr*^*−/−*^, and *Oxt*^*−/−*^;*Ptgfr*^*−/−*^ females were 20.5 ± 1.4 g, 20.2 ± 1.1 g, and 20.7 ± 0.6 g, respectively (n = 3 to 5, 11 to 14 weeks old).

Mice were housed in rooms with controlled temperature (25 ± 2°C) under a 10-hour light/14-hour dark cycle (lights on at 5:30 am to 7:30 pm). Females (8 to 29 weeks old) were mated with male C57BL/6J mice (Japan SLC, Shizuoka, Japan) during the dark phase. Females were separated from males the next morning. The morning when a vaginal plug was observed was defined as gestation day (GD) 0.5. The pregnant females were individually housed from GD 17.5 on. From the day before expected birth (GD 18.5), pregnant females were observed every 6 hours and the number of pups at each time was recorded. Initiation of parturition was determined as the time that a female delivered the first pup. The uterus of each female was checked by opening the abdominal cavity 24 hours after birth of the first pup. All of the pups that remained in the uterus of *Oxtr*^*−/−*^;*Ptgfr*^*+/−*^, *Oxtr*^*−/−*^;*Ptgfr*^*−/−*^, and *Oxt*^*−/−*^;*Ptgfr*^*−/−*^ died at that time.

### RNA extraction and reverse transcription reaction

Total RNA from uterine horns of *Oxtr*^*−/−*^ and WT was isolated with TRIzol reagent (Invitrogen, Waltham, MA) and digested with DNase I (Takara Bio, Shiga, Japan) to prevent genomic DNA contamination. Ten micrograms of DNase I–treated RNA was then reverse transcribed by using SuperScript II reverse transcription (Invitrogen, Waltham, MA) and oligo(dT) primer in a 97-μL reaction volume according to the manufacturer’s instructions. Nondiluted first-strand cDNA solution was used for quantitative real-time PCR (qPCR).

### qPCR

qPCR was conducted to measure the relative mRNA expression levels of the oxytocin receptor, PGF_2*α*_ receptor, connexin 43, COX-1, COX-2, and PGE_2_ receptors 1 to 4 using a DNA Engine Opticon system (MJ Japan, Tokyo, Japan). Mouse ribosomal protein large P0 was used as an internal control. The assay was performed in a 20-μL reaction volume containing DyNAmo SYBR Green qPCR master mix (Finnzymes, Espoo, Finland), 1 μL of cDNA solution, and 12.5 pmol of each of gene-specific primer. The primers used are shown in Table 1. Each reaction was performed in duplicate. Expression of mRNA for target genes was normalized relative to that the internal control (ribosomal protein large P0) mRNA using the ΔΔCT method (20). Amplification efficiency of primer sets on the uterine contraction-associated genes was between 89% and 99% of that on the internal control. A melt curve analysis was performed after each PCR run to ensure that a single product was amplified.

**Table 1.** PCR Primer Sequences Used for qPCR

Gene Name	GenBank Accession No.	Forward Primer (5′→3′)	Reverse Primer (5′→3′)
Oxytocin receptor	NM_001081147	TTCTTCGTGCAGATGTGGAG	AGGACGAAGGTGGAGGAGTT
PGF_2*α*_ receptor	NM_008966	GCTCTTGGTGTTTCCTTCTCG	ACAGCCTTCCGTAGCAGAAT
Connexin 43	NM_010288	CCCGAACTCTCCTTTTCCTT	GGGCACAGACACGAATATGA
COX-1	NM_008969	CCAGAACCAGGGTGTCTGTGT	GTAGCCCGTGCGAGTACAATC
COX-2	NM_011198	TGAGCACAGGATTTGACCAG	CAATGTTCCAGACTCCCTTGA
PGE_2_ receptor 1	NM_013641	TGGGTCGCTACGAGTTACAG	TGTGGCTGAAGTGATGGATG
PGE_2_ receptor 2	NM_008964	AATGCGCTCAGTCCTCTGTT	CAGCCCCTTACACTTCTCCAATGA
PGE_2_ receptor 3	NM_001359745	TGACCTTTGCCTGCAACCTG	AACAGACGGACAGCACACAC
PGE_2_ receptor 4	NM_001136079	CAGCTCCTTCCTCATCCTTG	CAGATGAGCACCACCAGAGA
Ribosomal protein large P0	NM_007475	ATAACCCTGAAGTGCTCGACAT	GGGAAGGTGTACTCAGTCTCCA

### Injection of RU486

*Ptgfr* ^*−/−*^, *Oxtr*^*−/−*^;*Ptgfr*^*−/−*^, and *Oxt*^*−/−*^;*Ptgfr*^*−/−*^ were injected subcutaneously with the progesterone receptor antagonist RU486 (Sigma-Aldrich, St. Louis, MO) (225 μg in 100 μL of peanut oil) or a vehicle on GD 19.0.

### Measurement of the plasma concentration of progesterone

Trunk blood was collected by decapitation on GD 17.0 and GD 19.0. Plasma samples were obtained by centrifugation. The plasma concentration of progesterone was measured in duplicate by an RIA (21) (Coat-A-Count progesterone kit, Siemens Healthcare Diagnostic Products, Murburg, Germany).

### Histological analysis of the cervix

Uterine cervixes of WT, *Oxtr*^*−/−*^, *Ptgfr*^*−/−*^, and *Oxtr*^*−/−*^;*Ptgfr*^*−/−*^ were isolated and fixed in 4% paraformaldehyde. The cervixes were frozen in OCT compound (Sakura Finetek Japan, Tokyo, Japan) and stored at −80°C. Coronal cervix sections were cut at 10 μm with a cryostat. The sections were assessed for collagen fibers by Elastica-Masson staining (blue stain). Muscle tissues and nuclei are stained red and deep red, respectively.

### Statistical analysis

Data are expressed as means + SEM. Data were analyzed by one-way ANOVA followed by a Tukey–Kramer posttest and Fisher exact probability test. *P* < 0.05 was considered statistically significant.

## Results

### Downregulation of COX-2 and connexin 43 expression but not PGF_2*α*_ receptor expression during parturition in the *Oxtr*^*−/−*^ uterus

**Figure 1.**
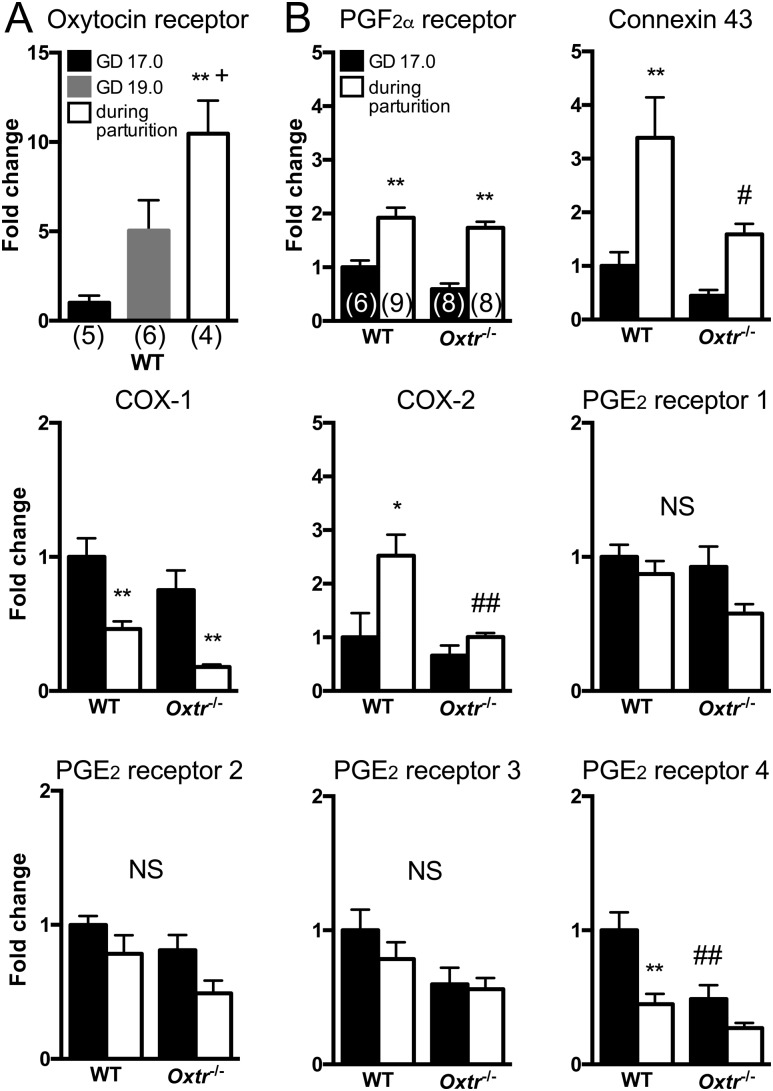
Fold change in expression of uterine contraction-associated genes in WT and *Oxtr*^−/−^ uteri. (A) qPCR analysis for oxytocin receptor expression in WT on GD 17.0, GD 19, and during parturition. (B) Real-time PCR analysis for PGF_2*α*_ receptor, connexin 43, COX-1, COX-2, and PGE_2_ receptors 1 to 4 in WT and *Oxtr*^−/−^ on GD 17.0 and during parturition. Fold change was normalized to the value of WT on GD 17.0. Data were analyzed by one-way ANOVA followed by a Tukey–Kramer posttest. Values in parentheses are the number of samples used per group. *P < 0.05, **P < 0.01 compared with GD 17.0; ^+^*P* < 0.05 compared with GD 19.0; ^#^*P* < 0.05, ^##^*P* < 0.01 compared with corresponding groups of WT. NS, not significant.

To clarify potential mechanisms for compensation of oxytocin receptor gene deficiency, we investigated whether expression of uterine contraction-associated genes changes in *Oxtr*^−/−^. We first confirmed the expression of mRNA for the oxytocin receptor in the WT uterus on GD 17.0 and GD 19.0 and during parturition. Oxytocin receptor expression during parturition was significantly increased compared with that on GD 17.0 and GD 19.0 (Fig. 1A). The expression of mRNA for the PGF_2*α*_ receptor, connexin 43, COX-1, COX-2, and PGE_2_ receptors 1 to 4 in the uterus was next examined on GD 17.0 and during parturition. PGF_2*α*_ receptor, connexin 43, and COX-2 expression levels were significantly increased in the WT uterus during parturition. COX-1 and PGE_2_ receptor 4 expression levels were significantly decreased in WT during parturition. PGE_2_ receptor 1, PGE_2_ receptor 2, and PGE_2_ receptor 3 expression levels did not show significant changes. In *Oxtr*^−/−^, we found that connexin 43 and COX-2 expression levels during parturition were not significantly increased compared with those on GD 17.0 and that the expression levels of these two genes during parturition were significantly lower than those in WT. The expression level of the PGE_2_ receptor 4 on GD 17.0 in *Oxtr*^−/−^ was significantly lower than that in WT. The expression levels of the PGF_2*α*_ receptor and COX-1 on GD 17.0 and during parturition were not significantly different between the genotypes (Fig. 1B). These results suggest that oxytocin receptor signaling upregulated the expression of connexin 43 and COX-2 during parturition, whereas upregulation of PGF_2*α*_ receptor expression during parturition is independent of oxytocin receptor signaling. We speculate that a myometrial action of PGF_2*α*_ receptor compensates for that of the oxytocin receptor.

### Impairment of decline in plasma progesterone concentration during late pregnancy and abnormal parturition in *Oxtr*^*−/−*^;*Ptgfr*^*−/−*^ and *Oxt*^*−/−*^;*Ptgfr*^*−/−*^ as in *Ptgfr*^*−/−*^

**Figure 2.**
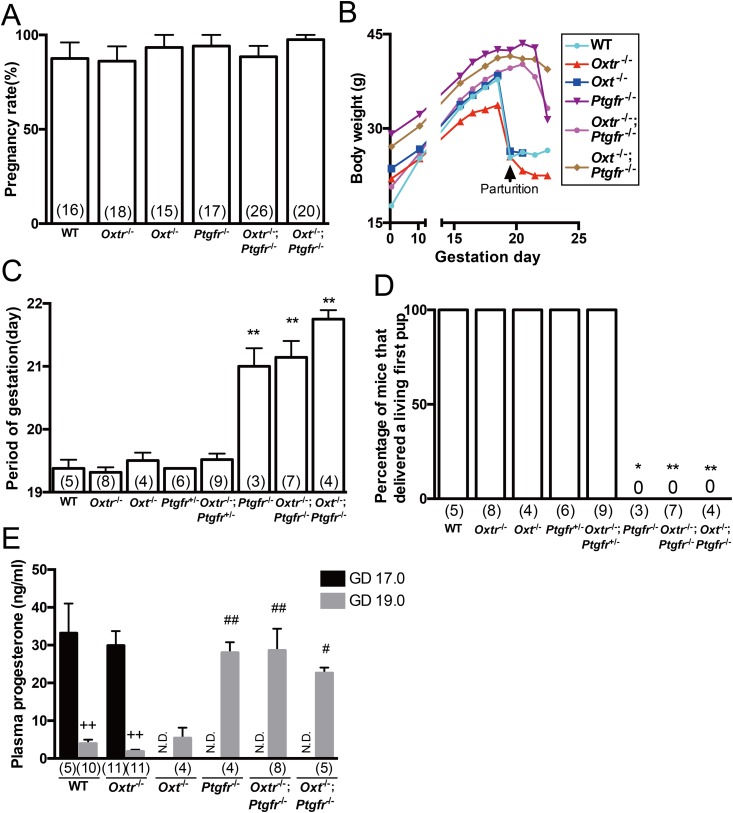
Failure of natural parturition in *Oxtr*^−/−^;*Ptgfr*^−/−^ and *Oxt*^−/−^;*Ptgfr*^−/−^. (A) Pregnancy rates of WT and mutant mice. (B) Changes of maternal body weight during pregnancy in WT and mutant mice. (C) Periods of gestation in WT and mutant mice. **P < 0.01 compared with WT mice. (D) Percentages of mice that delivered living pups in WT and mutant mice. *P < 0.05, **P < 0.01 compared with WT. (E) Plasma concentrations of progesterone in WT and mutant mice on GD 17.0 and GD 19.0. ^++^*P* < 0.01 compared with GD 17.0 of corresponding groups; ^#^*P* < 0.05, ^##^*P* < 0.01 compared with GD 19.0 of the WT group. Data were analyzed by (A, C, and E) one-way ANOVA followed by a Tukey–Kramer posttest or (D) Fisher exact probability test. Values in parentheses are the number of samples used per group. N.D., not determined.

To determine the cooperative role of the oxytocin/oxytocin receptor system and the PGF_2*α*_ receptor in the process of parturition, we generated *Oxtr*^*−/−*^;*Ptgfr*^*−/−*^ and *Oxt*^*−/−*^;*Ptgfr*^*−/−*^. We first compared the pregnancy rates in WT and mutant mice. In *Oxtr*^*−/−*^;*Ptgfr*^*−/−*^ and *Oxt*^*−/−*^;*Ptgfr*^*−/−*^, pregnancy rates were not significantly different compared with those in other genotypes (Fig. 2A). We next investigated the timing of parturition in WT and mutant mice. The body weights of WT, *Oxtr*^*−/−*^, and *Oxt*^*−/−*^ increased by GD 19.0 or GD 19.5. Then they delivered their pups and their body weights decreased. The body weights of *Oxtr*^*−/−*^;*Ptgfr*^*−/−*^ and *Oxt*^*−/−*^;*Ptgfr*^*−/−*^ also gradually increased through pregnancy. However, their body weight increased until GD 21.5 and then gradually decreased because of excretion or absorption of fetuses that died *in utero* (Fig. 2B). Their phenotype was similar to that of *Ptgfr*^*−/−*^. No normal parturition was observed in *Ptgfr*^*−/−*^, *Oxtr*^*−/−*^;*Ptgfr*^*−/−*^ and *Oxt*^*−/−*^;*Ptgfr*^*−/−*^. The periods of gestation were not significantly different among WT, *Oxtr*^*−/−*^, *Oxt*^*−/−*^, *Ptgfr*^*+/−*^, and *Oxtr*^*−/−*^;*Ptgfr*^*+/−*^, and the periods were ∼19.5 days. In *Ptgfr*^*−/−*^, *Oxtr*^*−/−*^;*Ptgfr*^*−/−*^, and *Oxt*^*−/−*^;*Ptgfr*^*−/−*^, the periods of gestation were significantly longer than that in WT, and the periods were ∼21.0 days (Fig. 2C). All of the WT, *Oxt*^*−/−*^, *Oxtr*^*−/−*^, *Ptgfr*^*+/−*^, and *Oxtr*^*−/−*^;*Ptgfr*^*+/−*^ were able to deliver a living first pup, whereas none of the *Ptgfr*^*−/−*^, *Oxtr*^*−/−*^;*Ptgfr*^*−/−*^, and *Oxt*^*−/−*^;*Ptgfr*^*−/−*^ was able to deliver their pups alive (Fig. 2D). We then examined the plasma concentrations of progesterone on GD 17.0 and GD 19.0. In WT and *Oxtr*^*−/−*^, progesterone levels on GD 19.0 were significantly lower than those on GD 17.0. Progesterone levels on GD 19.0 were not significantly different between WT and *Oxtr*^*−/−*^ or *Oxt*^*−/−*^. In *Ptgfr*^*−/−*^, *Oxtr*^*−/−*^;*Ptgfr*^*−/−*^, and *Oxt*^*−/−*^;*Ptgfr*^*−/−*^, progesterone levels on GD 19.0 were not significantly different from that in WT on GD 17.0 and the levels were significantly higher than that in WT on GD 19.0 (Fig. 2E). These results suggest that persistent production of progesterone leads to failure of normal parturition in *Oxtr*^*−/−*^;*Ptgfr*^*−/−*^ and *Oxt*^*−/−*^;*Ptgfr*^*−/−*^ as in *Ptgfr*^*−/−*^.

### Delivering live pups by inhibition of progesterone receptor activity in *Oxtr*^*−/−*^;*Ptgfr*^*−/−*^ and *Oxt*^*−/−*^;*Ptgfr*^*−/−*^

**Figure 3.**
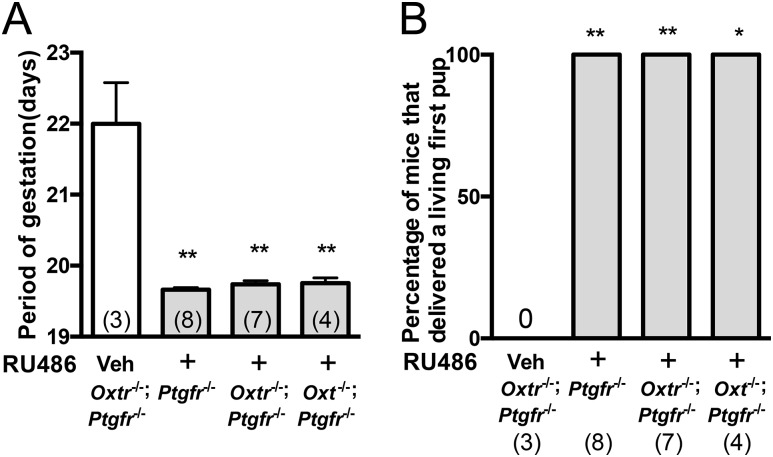
Successful parturition onset in *Oxtr*^−/−^;*Ptgfr*^−/−^ and *Oxt*^−/−^;*Ptgfr*^−/−^ with injection of the progesterone antagonist RU486. (A) Period of gestation in mutant mice administered RU486 or a vehicle. (B) Percentage of mice that delivered living pups after injection of RU486 in mutant mice. *P < 0.05, **P < 0.01 compared with the vehicle-injected group of *Oxtr*^−/−^;*Ptgfr*^−/−^. Data were analyzed by one-way ANOVA followed by (A) a Tukey–Kramer posttest or (B) Fisher exact probability test. Values in parentheses are the number of samples used per group. Veh, vehicle injection; +, RU486 injection.

To induce a decline in progesterone activity during parturition, we injected the progesterone receptor antagonist RU486 on GD 19.0 in *Ptgfr*^*−/−*^, *Oxtr*^*−/−*^;*Ptgfr*^*−/−*^, and *Oxt*^*−/−*^;*Ptgfr*^*−/−*^. In RU486-injected *Ptgfr*^*−/−*^, *Oxtr*^*−/−*^; *Ptgfr*^*−/−*^, and *Oxt*^*−/−*^;*Ptgfr*^*−/−*^, the periods of gestation were significantly shorter than that in vehicle-injected *Oxtr*^*−/−*^;*Ptgfr*^*−/−*^ and they were ∼19.5 days. There were no significant differences between the periods of gestation in RU486-injected *Ptgfr*^*−/−*^, *Oxtr*^*−/−*^;*Ptgfr*^*−/−*^, and *Oxt*^*−/−*^;*Ptgfr*^*−/−*^ (Fig. 3A). All of the RU486-injected *Ptgfr*^*−/−*^, *Oxtr*^*−/−*^;*Ptgfr*^*−/−*^, and *Oxt*^*−/−*^;*Ptgfr*^*−/−*^ were able to deliver a living first pup (Fig. 3B). These results suggest that activation of both the uterine PGF_2*α*_ receptor and the oxytocin/oxytocin receptor system are not essential for the process of parturition onset after withdrawal of plasma progesterone in mice.

### Prolonged parturition in *Oxtr*^*−/−*^;*Ptgfr*^*−/−*^ and *Oxt*^*−/−*^;*Ptgfr*^*−/−*^

**Figure 4.**
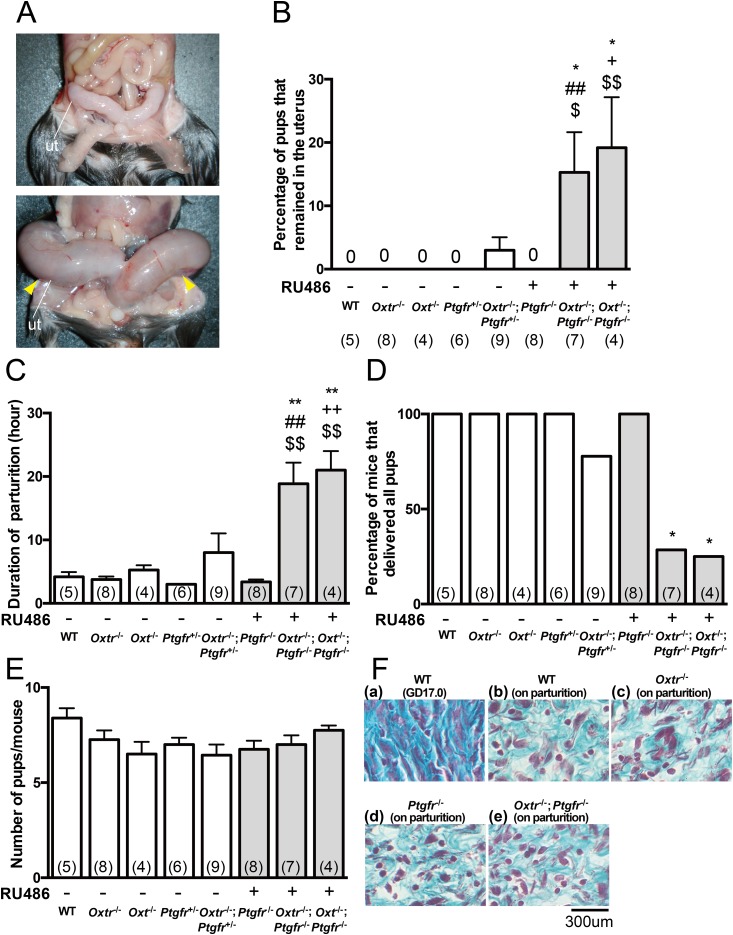
Prolonged parturition in *Oxtr*^−/−^;*Ptgfr*^−/−^ and *Oxt*^−/−^;*Ptgfr*^−/−^. (A) Photographs showing uteri of RU486-injected *Ptgfr*^−/−^ (upper) and *Oxtr*^−/−^;*Ptgfr*^−/−^ (lower) 24 h after the onset of parturition. Yellow arrowheads point to pups that remained in the uterus. Ut, uterus. (B) Percentages of pups that remained in the uterus in WT and mutant mice 24 h after the onset of parturition. *P < 0.05 compared with WT; ^##^*P* < 0.01 compared with *Oxtr*^−/−^; ^+^*P* < 0.05 compared with *Oxt*^−/−^; ^$^*P* < 0.05, ^$$^*P* < 0.01 compared with *Ptgfr*^−/−^. (C) Durations of parturition in WT and mutant mice. **P < 0.01 compared with WT; ^##^*P* < 0.01 compared with *Oxtr*^−/−^; ^++^*P* < 0.01 compared with *Oxt*^−/−^; ^$$^*P* < 0.01 compared with *Ptgfr*^−/−^. (D) Percentages of mice that delivered all pups in WT and mutant mice during 24 h. *P < 0.05 compared with WT. (E) Total number of pups (sum of pups delivered and pups that remained in the uterus). (F) Elastica-Masson staining of midcervical transverse sections of WT and mutant mice. (a) WT on GD 17.0 (n = 2), (b) WT during parturition (n = 2), (c) *Oxtr*^*−/−*^ during parturition (n = 5), (d) RU486-injected *Ptgfr*^−/−^ during parturition (n = 3), (e) RU486-injected *Oxtr*^−/−^;*Ptgfr*^−/−^ during parturition (n = 3). Data were analyzed by (B, C, and E) one-way ANOVA followed by a Tukey–Kramer posttest or (D) Fisher exact probability test. Values in parentheses are the number of samples used per group. −, nontreatment; +, RU486 injection.

We next examined the processes of parturition in detail in WT and mutant mice. We found that *Oxtr*^*−/−*^;*Ptgfr*^*+/−*^ and RU486-injected *Oxtr*^*−/−*^;*Ptgfr*^*−/−*^ and *Oxt*^*−/−*^;*Ptgfr*^*−/−*^ had partial abdominal swelling at 24 hours after the onset of parturition (Fig. 4A). All of the pups that remained in the uterus were dead at 24 hours after the onset of parturition. At 24 hours after the onset of parturition, 3.0% of the pups in *Oxtr*^*−/−*^;*Ptgfr*^*+/−*^, 15.3% of the pups in RU486-injected *Oxtr*^*−/−*^;*Ptgfr*^*−/−*^, and 19.2% of the pups in RU486-injected *Oxt*^*−/−*^;*Ptgfr*^*−/−*^ remained in the uterus. Alternatively, none of the WT, *Oxtr*^*−/−*^, *Oxt*^*−/−*^, *Ptgfr*^*+/−*^, and RU486-injected *Ptgfr*^*−/−*^ showed an abnormality. The percentages of pups that remained in the uterus in RU486-injected *Oxtr*^*−/−*^;*Ptgfr*^*−/−*^ and *Oxt*^*−/−*^;*Ptgfr*^*−/−*^ were significantly higher than those in WT, *Oxtr*^*−/−*^, *Oxt*^*−/−*^, and RU486-injected *Ptgfr*^*−/−*^ (Fig. 4B). The duration of parturition in RU486-injected *Ptgfr*^*−/−*^ was not significantly different from that in WT. Alternatively, the durations of parturition in RU486-injected *Oxtr*^*−/−*^;*Ptgfr*^*−/−*^ and *Oxt*^*−/−*^;*Ptgfr*^*−/−*^ were significantly longer than those in WT, *Oxtr*^*−/−*^, *Oxt*^*−/−*^, and RU486-injected *Ptgfr*^*−/−*^ (Fig. 4C). The percentages of mice that completed their parturition during a period of 24 hours were 100% for WT, *Oxtr*^*−/−*^, *Oxt*^*−/−*^, *Ptgfr*^*+/−*^, and RU486-injected *Ptgfr*^*−/−*^, 75% for *Oxtr*^*−/−*^;*Ptgfr*^*+/−*^, and ∼20% for RU486-injected *Oxtr*^*−/−*^;*Ptgfr*^*−/−*^ and *Oxt*^*−/−*^;*Ptgfr*^*−/−*^ (Fig. 4D). The litter sizes were not significantly different between genotypes (Fig. 4E). It is known that ripening of the uterine cervix via collagen reorganization in late pregnancy plays a crucial role in prevention of prolonged labor (22). On GD 19.0, cervixes showed a loose array of disordered collagen fibers, whereas cervixes on GD 17.0 exhibited a denser and more heavily stained matrix of collagen fibers. Morphology of cervixes during parturition in *Oxtr*^*−/−*^, RU486-injected *Ptgfr*^*−/−*^, and RU486-injected *Oxtr*^*−/−*^;*Ptgfr*^*−/−*^ was similar to that in WT during parturition (Fig. 4F). These results suggest that both the PGF_2*α*_ receptor and the oxytocin/oxytocin receptor system have critical roles in uterine contraction for delivery but not in reconstruction of the uterine cervix.

## Discussion

Our experiments showed that oxytocin receptor signaling is necessary for induction of COX-2 and connexin 43 expression during parturition and that both oxytocin/oxytocin receptor signaling and PGF_2*α*_ receptor signaling are major components for successful parturition. In the current study, it was found that expression of the oxytocin receptor, PGF_2*α*_ receptor, COX-2, and connexin 43 in uteri increased during parturition, whereas the expression of COX-1 and PGE_2_ receptors 1 to 4 decreased or did not change during parturition compared with that in WT on GD 17.0. Alternatively, an increase in the expression of COX-2 and connexin 43 during parturition was not observed in the uteri of *Oxtr*^−/−^. The importance of COX-2 and connexin 43 in parturition has been suggested in previous reports. It has been shown that inflammation induced COX-2 expression in the uterus and that inhibition of the enzymatic activity of COX-2 prevented inflammation-mediated preterm labor (12). Connexin 43 contributes to the induction of physical and biochemical connectivity among myometrial cells and to the formation of extensive waves of depolarization and contraction over large areas of the uterus (23). Decline of blood progesterone concentration is critical for the onset of parturition in rodents. There is evidence that the progesterone receptor directly suppresses the expression of COX-2 and connexin 43. Previous studies showed that the progesterone receptor inhibited induction of COX-2 expression via both direct interaction with nuclear factor *κ*B (NF-*κ*B) (24) and induction of expression of the NF-*κ*B inhibitor I*κ*B*α* in myometrial cells (25). The progesterone receptor also binds to the promoter region of connexin 43 through p54nrb (non-POU domain–containing, octamer binding protein) and directly suppresses connexin 43 expression (26). Alternatively, there is evidence that the progesterone receptor indirectly suppresses oxytocin receptor expression. It was reported that ZEB1 and ZEB2, zinc finger E-box binding homeobox proteins, suppress oxytocin receptor transcription in the uterus until parturition. A decline of progesterone concentration induces downregulation of ZEB1 and ZEB2 at term. This downregulation induces oxytocin receptor transcription at term (27). There is no evidence that the progesterone receptor directly or indirectly suppresses expression of *Ptgfr*. In the current study, upregulation of COX-2 and connexin 43 transcription was impaired in the uterus of *Oxtr*^−/−^ during parturition, whereas PGF_2*α*_ receptor expression was not. Because *Oxtr*^−/−^ showed a progesterone concentration similar to that in WT on GD 19.0, the lower induction of COX-2 and connexin 43 expression is not caused by insufficient progesterone decline on term. The oxytocin receptor, COX-2, and connexin 43 are all expressed in the rodent myometrium in late pregnancy (17, 28, 29). It was reported that the oxytocin receptor activated the MAPK pathway and NF-*κ*B via the protein kinase A pathway. Oxytocin increased COX-2 expression and prostaglandin synthesis via MAPK *in vitro* (30). A study using ovariectomized *Ptgfr*^*−/−*^ showed that COX-2 expression increased after induction of oxytocin receptor expression in the uterus (28). Oxytocin receptor signaling also enhanced connexin 43 protein expression via activated NF-*κ*B *in vitro* (31). It is likely that the MAPK pathway and the protein kinase A–NF-*κ*B pathway via oxytocin receptor signaling enhance COX-2 and connexin 43 expression during parturition after the decline of progesterone concentration. To our knowledge, our findings provide the first evidence that oxytocin receptor signaling is essential for enhancement of COX-2 and connexin 43 expression during parturition.

Deletion of the PGF_2*α*_ receptor gene or COX-1 gene (a major gene responsible for the synthesis of PGF_2*α*_ in luteolysis) in mice resulted in a deficit of luteolysis at term, and progesterone concentration did not decline during late pregnancy in these deficient mice (9, 32). PGF_2*α*_ receptor signaling induces downregulation of blood progesterone concentrations by inhibition of progesterone biosynthesis in the corpora lutea (33). Alternatively, it was reported that mice with double knockout of the genes for COX-1/oxytocin showed normal onset of parturition. *Oxtr* expression in the corpora lutea declined in late pregnancy, and exogenous oxytocin infusion delayed the decline of progesterone concentration and onset of parturition (32). Those studies suggest that oxytocin has a luteotrophic effect in pregnancy, although *Oxt*^*−/−*^ and *Oxtr*^*−/−*^ showed normal parturition and a serum progesterone decline similar to that in WT mice. In the current study, *Oxtr*^*−/−*^;*Ptgfr*^*−/−*^ and *Oxt*^*−/−*^;*Ptgfr*^*−/−*^ showed a plasma progesterone concentration similar to that in *Ptgfr*^*−/−*^ on GD 19.0. Considering the previous study, oxytocin receptor signaling appears to have the opposite action of PGF_2*α*_ in luteolysis in late pregnancy. However, oxytocin and oxytocin receptor gene deficiencies were not able to restore the decline of progesterone concentration and normal onset of parturition in complete blocking of PGF_2*α*_ receptor signaling by gene deficiency, unlike COX-1 gene deficiency. These findings suggested that a luteotrophic action of oxytocin/oxytocin receptor signaling in the corpora lutea in late pregnancy is negligible.

In the current study, *Oxtr*^*−/−*^;*Ptgfr*^*−/−*^ and *Oxt*^*−/−*^;*Ptgfr*^*−/−*^ administered RU486 were capable of starting parturition. Uteri in both *Oxtr*^*−/−*^ and *Ptgfr*^*−/−*^ at late pregnancy lost contractile responses to oxytocin and PGF_2*α*_, respectively (3, 9). These results suggest that there are certainly other uterine contractile mechanisms for the onset of parturition other than oxytocin/oxytocin receptor signaling and PGF_2*α*_ receptor signaling. Inhibition of the enzymatic activity of COX-2 postponed the onset of parturition in mice (28, 34). PGE_2_ has been reported to have potent uterotonic activity in the periparturient uterus. PGE_2_ receptors 1 and 3 were expressed in the myometrium at term of parturition, and PGE_2_ receptor 3 induced contractility in the human pregnant myometrium (13). Alternatively, it is known that PGE_2_ plays an essential role in cervical ripening during term. Downregulation of 15-prostaglandin dehydrogenase, which inactivates PGE_2_, is critical for cervix ripening at term (35). COX-2 expression increased after induction of oxytocin receptor expression in the uterus during parturition (28) and COX-2 expression was not induced in the uterus of *Oxtr*^−/−^ during parturition in this study. These results suggest that induction of COX-2 expression in the uterus at term is not essential to induce contractility for parturition onset. Further investigation is needed to find contractile factors responsible for the onset of parturition. Cervical ripening is important for normal onset and smooth progress of parturition. Impairment of cervical ripening caused failure of parturition onset (22). Alternatively, early cervical ripening caused preterm birth (35). In the current study, *Oxtr*^*−/−*^;*Ptgfr*^*−/−*^ administered RU486 showed cervical histology similar to that in WT, *Oxtr*^*−/−*^, and RU486-injected *Ptgfr*^*−/−*^ at term, suggesting that oxytocin/oxytocin receptor signaling and PGF_2*α*_ receptor signaling are not likely to contribute to cervical ripening.

*Oxtr* ^*−/−*^;*Ptgfr*^*−/−*^ and *Oxt*^*−/−*^;*Ptgfr*^*−/−*^ administered RU486 showed greatly prolonged parturition and their pups remained in the uterus. *Oxtr*^*−/−*^;*Ptgf*^*-+/−*^ showed prolonged parturition as did *Oxtr*^*−/−*^;*Ptgfr*^*−/−*^ and *Oxt*^*−/−*^;*Ptgfr*^*−/−*^, although the percentage of *Oxtr*^*−/−*^;*Ptgf*^*-+/−*^ showing prolonged parturition (22.2%) was lower than the percentages of *Oxtr*^*−/−*^;*Ptgfr*^*−/−*^ and *Oxt*^*−/−*^;*Ptgfr*^*−/−*^ (71.4% and 75.0%, respectively). These findings suggested that both oxytocin/oxytocin receptor signaling and PGF_2*α*_ receptor signaling play an important role in the progress of parturition. Sensitivity of uterine contraction to vasopressin increased at late pregnancy (7). Vasopressin induced uterine contraction not via the vasopressin receptor but via the oxytocin receptor in the mouse uterus in late pregnancy (3, 6). In the current study, all of the phenotypes in *Oxt*^*−/−*^;*Ptgfr*^*−/−*^ administered RU486 were similar to those of *Oxtr*^*−/−*^;*Ptgfr*^*−/−*^ administered RU486. These findings suggested that vasopressin did not compensate for oxytocin function via the oxytocin receptor and did not have a major contribution to the progress of parturition in mice.

**Figure 5.**
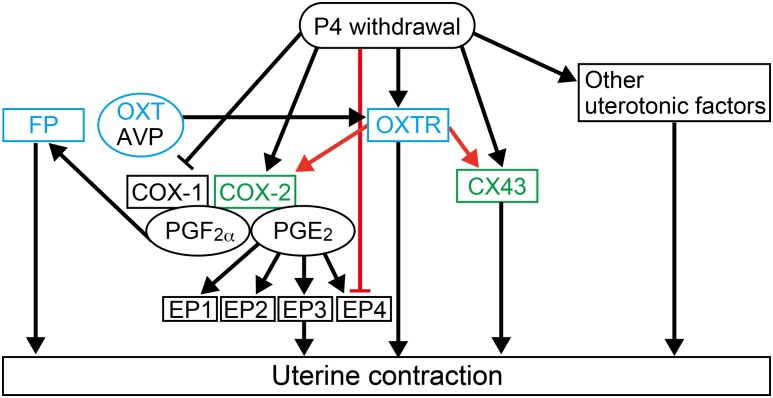
Schematic representation of the hierarchy for uterotonics in the progress of parturition. Blue indicates deficient mice that were used in this study; double deletion of the PGF_2*α*_*–*oxytocin system impaired the progress of parturition. Red arrows indicate novel pathways confirmed in the uterus in late pregnancy. Green indicates downregulated genes in the uterus of *Oxtr*^−/−^ during parturition. AVP, vasopressin; CX43, connexin 43; EP1, PGE_2_ receptor 1; EP2, PGE_2_ receptor 2; EP3, PGE_2_ receptor 3; EP4, PGE_2_ receptor 4; FP, PGF_2*α*_ receptor; OXT, oxytocin; OXTR, oxytocin receptor; P4, progesterone.

In humans, maternal serum progesterone concentrations do not vary significantly in the late third trimester of pregnancy, and there is no evidence of a fall in maternal plasma or uterine tissue progesterone at labor, unlike those of rodents (36). However, administration of RU486 can induce human labor at term (37). Additionally, some reports suggest the importance of “functional progesterone withdrawal” in initiation of human labor. Progesterone receptors are expressed as two protein isoforms. Progesterone receptor-A and progesterone receptor-B are structurally identical with the exception of N-terminal extension that is specific to progesterone receptor-B. This extended region possesses a transactivation activity that contributes to distinct cell- and promoter-specific transactivation properties of the two isoforms (38). At term in human labor, the progesterone receptor-A/progesterone receptor-B ratio increases due to increased progesterone receptor-A expression in the myometrium (39). Progesterone receptor-A represses the transcriptional activity of progesterone receptor-B in human myometrial cells (40). Additionally, the expression of progesterone receptor coactivators is decreased in the myometrium at term (41). In rodents, *Ptgfr*^*−/−*^ studies clearly indicated that a decline of maternal blood progesterone concentration via luteolysis in late pregnancy is essential for initiation of labor (9, 11). However, there is no genetic evidence that a decline of progesterone receptor activity is necessary for initiation of labor, because progesterone receptor-A–deficient female mice were infertile and progesterone receptor-B–deficient female mice had sustained pregnancy and gave birth to viable offspring (38, 42). RU486 binds to both progesterone receptors and the glucocorticoid receptor as an antagonist. There is no evidence that inhibition of the glucocorticoid receptor induces labor in humans (37, 43). In rodents, uterine-specific glucocorticoid receptor–deficient female mice had sustained pregnancy and were able to deliver their pups (44). All of these findings suggest that a decrease of progesterone receptor activity is a common event in humans and rodents for initiation of labor. A schematic representation of the hierarchy for uterotonics in the progress of parturition is shown in Fig. 5. Our results indicated that the expression of oxytocin receptor, PGF_2*α*_ receptor, COX-2, and connexin 43 is induced during parturition in mice. In humans, oxytocin and PGF_2*α*_ have been used to induce or augment labor in clinical practice (1, 8). The expression of oxytocin receptor, PGF_2*α*_ receptor, COX-2, and connexin 43 was upregulated at term in labor in the human myometrium (17, 45–47). Additionally, oxytocin upregulated the expression of COX-2 in human myometrial cells *in vitro* (30). These findings suggest that the hierarchy for uterotonics obtained from the current study in mice has similarity with the mechanism in humans. Our study can help to further understand the complex mechanisms of human parturition.

Preterm birth is the main cause of neonatal morbidity and mortality: it causes 25% to 50% of long-term neurologic impairment in children and ∼70% of neonatal deaths and 36% of infant deaths (48). It is recognized that an increase in uterine contractions and shortening and dilatation of the cervix are features of active parturition, although the processes of human parturition are not fully understood. Recent reports suggest that some tocolytic agents might be effective in women with preterm labor. A promising nonpeptide oxytocin receptor antagonist, GSK221149A (retosiban), is currently being used in a phase 3 clinical trial for treatment of spontaneous preterm labor (49). Selective PGF_2*α*_ receptor antagonists OBE022 and OBE002 are currently being used in a phase 2 clinical trial for pregnant women with preterm labor (50). Administration of retosiban to women with spontaneous preterm labor was associated with an increase of 8.2 days in time to delivery compared with that in women administered a placebo and significant reduction in preterm births (51). Perinatal survival rate for preterm infants dramatically increased week by week between 22 weeks and 31 weeks of gestation, indicating that a longer period of uterine quiescence with tocolytic reagents can contribute to a decrease in the risk (52). We showed that the oxytocin receptor and PGF_2*α*_ receptor have a complementary relationship in the process of parturition. Our results suggest that administration of an oxytocin receptor antagonist in combination with a PGF_2*α*_ receptor antagonist would be more effective for spontaneous preterm labor.

In conclusion, the oxytocin receptor is an upstream regulator of COX-2 and connexin 43 expression in the uterus during parturition, and both oxytocin/oxytocin receptor signaling and PGF_2*α*_ receptor signaling are major components for successful parturition. Our results thus indicate the transcriptional and functional hierarchy of uterotonics required for successful parturition.

## Additional Information

***Disclosure Summary:*** The authors have nothing to disclose.

***Data Availability:*** The datasets generated during and/or analyzed during the current study are not publicly available but are available from the corresponding author on reasonable request.

## Abbreviations:

COX: cyclooxygenase
GD: gestation day
NF-*κ*B: nuclear factor *κ*B
*Oxt*^−/−^: oxytocin-deficient mice
*Oxtr*^−/−^: oxytocin receptor–deficient mice
PGE_2_: prostaglandin E_2_
PGF_2*α*_: prostaglandin F_2*α*_
*Ptgfr*^*−/−*^: prostaglandin F_2*α*_ receptor–deficient mice
qPCR: quantitative real-time PCR
WT: wild-type
